# Eco-Friendly, High-Density Fiberboards Bonded with Urea-Formaldehyde and Ammonium Lignosulfonate

**DOI:** 10.3390/polym13020220

**Published:** 2021-01-10

**Authors:** Petar Antov, Viktor Savov, Ľuboš Krišťák, Roman Réh, George I. Mantanis

**Affiliations:** 1 Department of Mechanical Wood Technology, Faculty of Forest Industry, University of Forestry, 1797 Sofia, Bulgaria; victor_savov@ltu.bg; 2 Faculty of Wood Sciences and Technology, Technical University in Zvolen, T. G. Masaryka 24, 960 01 Zvolen, Slovakia; reh@tuzvo.sk; 3 Lab of Wood Science and Technology, Department of Forestry, Wood Sciences and Design, University of Thessaly, 43100 Karditsa, Greece; mantanis@uth.gr

**Keywords:** wood-based panels, high-density fiberboards, bio-adhesives, ammonium lignosulfonate, zero-formaldehyde emission

## Abstract

The potential of producing eco-friendly, formaldehyde-free, high-density fiberboard (HDF) panels from hardwood fibers bonded with urea-formaldehyde (UF) resin and a novel ammonium lignosulfonate (ALS) is investigated in this paper. HDF panels were fabricated in the laboratory by applying a very low UF gluing factor (3%) and ALS content varying from 6% to 10% (based on the dry fibers). The physical and mechanical properties of the fiberboards, such as water absorption (WA), thickness swelling (TS), modulus of elasticity (MOE), bending strength (MOR), internal bond strength (IB), as well as formaldehyde content, were determined in accordance with the corresponding European standards. Overall, the HDF panels exhibited very satisfactory physical and mechanical properties, fully complying with the standard requirements of HDF for use in load-bearing applications in humid conditions. Markedly, the formaldehyde content of the laboratory fabricated panels was extremely low, ranging between 0.7–1.0 mg/100 g, which is, in fact, equivalent to the formaldehyde release of natural wood.

## 1. Introduction

The growing need for sustainable products and the stringent legislative requirements related to the hazardous formaldehyde emissions from wood-based panels have boosted scientific and industrial interest in the production of eco-friendly, wood-based panels [1,2,3,4,5,6,7] and optimal utilization of the available lignocellulosic materials [8,9,10,11,12,13].

Fiberboards are wood-based panels produced by breaking down softwood and hardwood material into fibers, mixing them with wax and a formaldehyde-based thermosetting resin, such as urea-formaldehyde (UF), phenol formaldehyde, or melamine-urea formaldehyde, and forming panels by applying pressure and high temperature in a hot press [14,15]. Depending on the density, fiberboards can be classified into low-density fiberboards, with densities less than 400 kg·m^−3^, medium-density fiberboards, with densities ranging from 400 to 900 kg·m^−3^, and high-density fiberboards (HDF), with densities ranging from 900 to 1100 kg·m^−3^. HDF panels are one of the most widely used wood-based products worldwide, with a variety of end-uses, such as high-grade furniture, laminate flooring, wall panels, shelves, and door skins. High-density fiberboards are denser than particleboards, and can be even denser than ordinary plywood; this characteristic broadens their application. HDF panels have many advantages, such as having a smoother surface, easier machinability, and increased strength. Resistance to direct screw withdrawal is relevant only for those fiberboard products used in furniture and cabinetmaking. Such panels are ideal for use as substrates for thin overlays in indoor conditions [16,17,18]. Another important advantage of HDF panels is the utilization of small-sized, low-quality wood, which is otherwise used mainly for energy purposes [19,20,21].

Traditional synthetic adhesives used in the production of wood-based panels are based on fossil-derived constituents, mainly urea, melamine, and phenol [22,23,24,25]. At present, about 95% of total wood adhesives used in the production of wood-based panels are formaldehyde-based resins [25]. UF resins dominate the global market, accounting for about 85% of the amino resins produced worldwide, with an estimated annual production of 11 million tons/year [2,25,26]. UF resins are thermosetting resins, the product of a reaction between urea and formaldehyde. These resins have been widely used in the production of engineered wood-based panels as a result of their numerous advantages, e.g., low press temperatures, short press times, excellent strength properties, chemical versatility, and a relatively low price [23,24,25,26,27,28,29,30]. The main drawback of UF resins, apart from their lower water resistance in comparison with phenol and melamine formaldehyde resins [31], is the release of hazardous volatile organic compounds (VOC) and free formaldehyde from the final UF-bonded products, especially with indoor use [32,33,34].

Formaldehyde (HCHO, CAS No. 50-00-0) is associated with a number of significant environmental issues [35] and serious human health hazards, such as skin and respiratory tract irritation, skin sensitization, nausea, genotoxicity, and cancer [36,37,38]. In 2004, formaldehyde was reclassified from “probable human carcinogen” to “known human carcinogen” (Group 1) by the International Agency for Research on Cancer [39]. Since then, the formaldehyde limit values have steadily been lowered, which has led to the development of less toxic, low-emission, eco-friendly, wood-based panels, where conventional synthetic resins have been partially or completely replaced by sustainable, bio-based adhesives [40,41,42,43,44,45,46,47] or by adding formaldehyde scavengers to adhesive formulations, such as urea [48], sodium metabisulfite (Na_2_S_2_O_5_), ammonium bisulfite ((NH_4_)HSO_3_), other ammonium-containing agents [49,50], Al_2_O_3_ nanoparticles [51], etc. Various natural compounds, such as wood bark flours [32,52,53,54] and tannins [55,56,57], have also been successfully used to reduce the free formaldehyde content of wood panels.

Different renewable biomass resources, such as proteins [58,59,60], starch [61,62], tannins [63,64,65], and lignin [9,66,67,68,69] have been used as sustainable raw materials for the development of bio-based adhesives for engineered wood panels.

Lignin is a polyaromatic amorphous macromolecule and the second most abundant organic material after cellulose [70,71]. It is considered to be low-value waste or a byproduct, e.g., of the pulp and paper industries, which generate approximately 50–70 million tons of lignin annually [72]. With the increased production of biofuels, it is estimated that this number will reach 225 million tons per year by 2030 [72]. The enhanced scientific and industrial interest in lignin valorization is due to its abundance, renewability, and potential for providing an economically and environmentally sustainable alternative raw material for the production of value-added products, including adhesives for wood-based panels [4,43,73]. The application of lignin in the formulation of adhesives is primarily due to its polyphenolic structure. Thus, lignin may be used in lignin-phenol-formaldehyde resins, where it is applied to partially replace phenol [6,74,75]. Additional chemical modification of lignin, such as phenolation and methylolation, can be applied to increase the lignin reactivity to formaldehyde [1,75,76].

Technical lignins are obtained by different pulping technologies, but only lignosulfonates, which are water-soluble polyelectrolytes derived as byproducts of the sulfite processes, are available in large quantities, with an estimated annual production of approximately 800,000 tons [77,78,79,80]. Lignosulfonates contain a large number of functional groups, resulting in excellent surface activity; ammonium lignosulfonates (ALS) have been shown to be the most suitable ones for adhesive applications due to their large number of hydroxyl groups [75,81]. Another important advantage of ALS is their solubility in a number of organic solvents, unlike sodium, calcium, and magnesium salts, which are only soluble in water [82].

The aim of this research work is to investigate the potential of producing eco-friendly HDF panels from hardwood fibers, bonded with UF resin and a novel ALS adhesive, in order to reach the European standard requirements.

## 2. Materials and Methods

Industrially produced fibers obtained in factory conditions through the Asplund-method, using a L46 Defibrator (Valmet, Stockholm,, Sweden), were used in this work. The fibers were supplied by the factory of Welde Bulgaria AD (Troyan, Bulgaria). The fibers were obtained by the thermo-mechanical defibration of wood chips with dimensions (length) from 5 to 30 mm, subjected to steam treatment at 0.8 MPa steam pressure and 170 °C temperature. The industrial wood fibers had a bulk density of 29 kg·m^−3^ and a pulp freeness of 11° SR (Schopper Riegler test). The fibers were composed of the hardwood species European beech (*Fagus sylvatica* L.) and Turkey oak (*Quercus cerris* L.) at a ratio of 2:1, and were oven dried to a moisture content of 6.2%. The fibers had lengths from 1116 µm to 1250 µm (factory data).

Urea-formaldehyde resin with a solid content of 64% and a molar ratio (MR) of 1.16 was provided by Kastamonu Bulgaria AD (Gorno Sahrane, Bulgaria).

Commercial ammonium lignosulfonate (D-947L) was supplied by Borregaard (Sarpsborg, Norway). The ALS (CAS No. 8061-53-8) adhesive had the following characteristics: ammonium content—4.1%, sodium content—0.1%, total sulfur content—6.8%, high performance liquid chromatography (HPLC) sugars—20%, pH—4.5%, total solids content—48.6%, specific gravity—1.220, boiling point 104 °C, evaporation rate 0.4, vapor pressure 14.2 mmHg, and viscosity (cps)—400 at 25 °C.

Under laboratory conditions, the HDF panels were produced with dimensions of 400 × 400 mm^2^, a thickness of 6 mm, and a target density of 910 kg·m^−3^. The adhesive formulation was comprised of UF resin at 3% and three addition levels of ALS (6%, 8% and 10%), based on the weight of fibers. The UF resin was used at 50% concentration. Urea at 3% based on the dry resin at 50% concentration was used as a formaldehyde scavenger. Ammonium sulfate ((NH_4_)_2_SO_4_, CAS No. CAS 7783-20-2) at 1.5% based on the dry UF resin and 2% based on dry D-947L at 30% concentration was used as a hardener.

A control panel was fabricated with 6% UF content, based on the dry fibers, and without ALS (HDF type 4). This resin addition level (6%) is typical for commercial HDF panels.

The manufacturing parameters of the laboratory-fabricated HDF panels are presented in Table 1.

The industrial wood fibers were mixed with the adhesive formulation in a high-speed laboratory glue blender (850 min^−1^). The hot pressing process was carried out using a single opening hydraulic press (PMC ST 100, Italy). The press temperature used was 200 °C. The press factor applied was 30 s·mm^−1^. The following four-stage pressing regime was used: in the first stage, the pressure was increased to 4.5 MPa for 20 s; in the second stage, the pressure was steadily decreased to 1.2 MPa; in the third stage, the pressure was decreased to 0.6 MPa. The fourth pressing stage was performed at a pressure of 1.8 MPa. After pressing, the fabricated composites were conditioned for 10 days at 20 ± 2 °C and 65% relative humidity.

**Figure 1:**
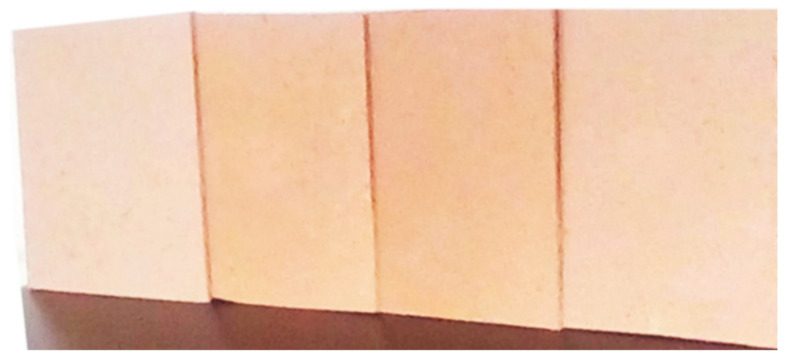
High-density fiberboards from industrial hardwood fibers bonded with UF resin and ammonium lignosulfonate; 910 kg·m^−3^ target density, 6 mm thickness and three addition levels of ammonium lignosulfonate (6%, 8%, 10%).

The physical and mechanical properties of the fabricated HDF panels (Figure 1) were tested according to European standards, namely EN 310, EN 317, EN 319, and EN 323 [83,84,85,86]. A precision laboratory balance Kern (Kern & Sohn GmbH, Balingen, Germany) with an accuracy of 0.01 g was used to determine the mass of the test specimens. The dimensions of the test pieces were measured using digital calipers with an accuracy of 0.01 mm. The physical properties (water absorption and thickness swelling) were measured after 24 h of immersion in water. The thickness swelling was assessed using the differences between the initial and final panel thicknesses, and the water absorption was determined using the difference in weight. The mechanical properties of the HDF panels were determined using a universal testing machine Zwick/Roell Z010 (Zwick/Roell GmbH, Ulm, Germany). For each parameter, eight HDF samples were used for testing.

The formaldehyde content of the laboratory-produced panels was tested in the laboratory of Kronospan Bulgaria EOOD (Veliko Tarnovo, Bulgaria) on four specimens in accordance with the commonly used Perforator method [87].

Variation and statistical analysis of the results was carried out by using the specialized software, QstatLab 6.0.

## 3. Results

### 3.1. Physical and Mechanical Properties

The results of the physical and mechanical properties of the HDF panels, comprised of industrial hardwood fibers bonded with UF resin and a novel ALS adhesive (D-947L), are presented in this part. The density of the laboratory-produced panels varied from 893 to 930 kg·m^−3^, which was close to the targeted value. The differences in the final density of the panels were significantly below 5%; thus, it did not have a significant effect on the mechanical and physical properties.

The physical properties of the laboratory-produced HDF panels, i.e., water absorption (WA) and thickness swelling (TS), were determined after 24 h of immersion in water. Both WA and TS are critical panel properties that are directly correlated with the dimensional stability of wood-based panels [22,24].

**Figure 2:**
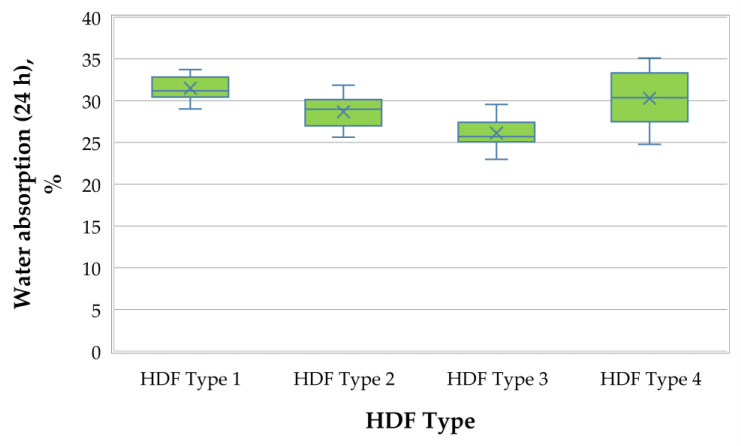
Water absorption (24 h) of HDF panels produced.

A graphical representation of the WA (24 h) of the laboratory-produced HDF panels is presented in Figure 2.

It was determined that increasing the ALS content from 6% to 10% resulted in decreased WA values of HDF panels from 31.5% to 26.1%, respectively; this means an average improvement of this property by 21%. The increased ALS addition resulted in a gradual decrease of WA values—a relative improvement of the WA by 10%, while increasing the ALS content by 2%. ALS has a pH of about 4.5, and the increased addition of ALS in the UF resin resulted in a decreased pH of the adhesive mixture. This acidic condition resulted in decreased fiber moisture absorption, and hence, improved the water resistance of the finished HDF panels. The increase in resistance of the UF resin modified by ALS was also caused by the decreased brittleness of the adhesive, which, in the case of the unmodified UF, causes the cured resin to crack and allow moisture to penetrate into the bonded product [88,89]. Only the HDF panel, fabricated with 3% UF resin and 6% ALS content, had higher WA values compared with the control HDF panel, produced with 6% UF resin and without ALS addition. The HDF panels, produced with 8% ALS and 10% ALS, had 1.06 times and 1.16 times lower WA values than the control panel, respectively.

WA is not a standardized technical property; nonetheless, according to the literature [24,90], the WA of common HDF panels typically varies between 30% and 45%. Thus, the HDF panels, produced under laboratory conditions from industrial wood fibers, bonded with a UF resin and an eco-friendly, formaldehyde-free ALS adhesive, exhibited comparable or better WA values compared with the industrially-produced HDF panels bonded with formaldehyde-based adhesives.

**Figure 3:**
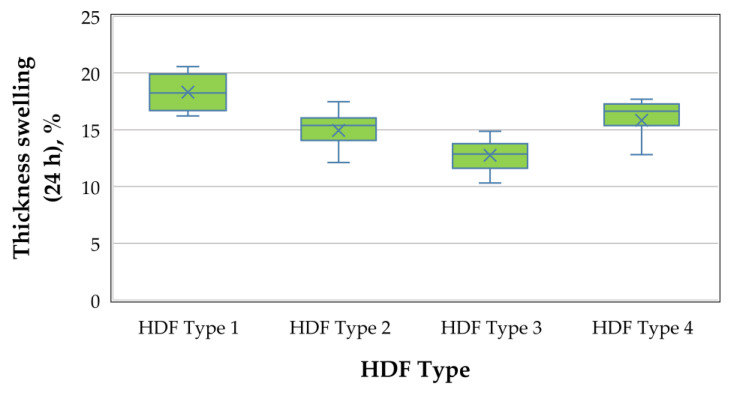
Thickness swelling of HDF panels produced.

A graphical representation of the TS (24 h) of the laboratory-produced HDF panels is shown in Figure 3.

As seen in Figure 3, TS of HDF panels bonded with ALS varied from 18.3% to 12.9%, i.e., increasing the ALS addition from 6% to 10% resulted in a 1.41 times improvement in TS values due to the decreased pH of the adhesive. All laboratory-produced panels had significantly better (lower) TS values than the standard requirement for application in humid conditions—25% [91]. The decreased pH of the adhesive may not be the only reason for the improvement in the TS values. Press temperature has a significant effect on the hygroscopic thickness swelling rate of HDF as well. The press temperature used in our research was 200 °C. It has been confirmed that the swelling rate increases as the HDF press temperature increases [92]. Only the HDF panel, produced with 3% UF resin and 6% ALS content, had higher TS values compared with the control panel (HDF type 4).

In terms of mechanical properties, the modulus of elasticity (MOE), bending strength (MOR), and internal bond (IB) strength of the laboratory-produced HDF panels were evaluated.

**Figure 4:**
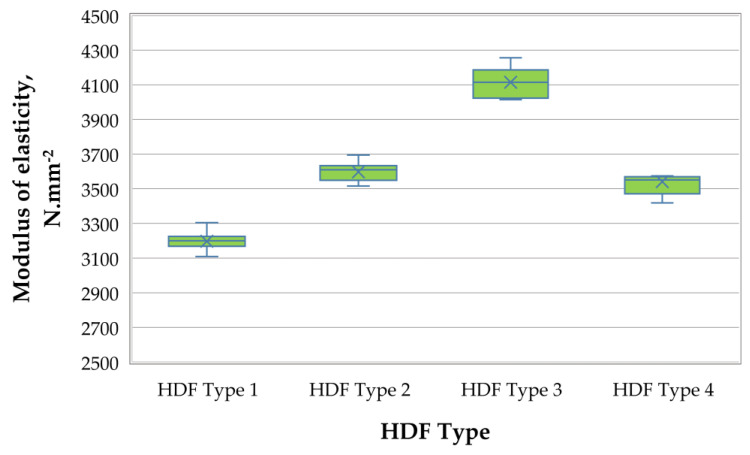
Modulus of elasticity (MOE) of HDF panels produced.

A graphic representation of the MOE of the laboratory-produced HDF panels is shown in Figure 4.

The MOE of fabricated HDF panels reached high values, ranging from 3197 to 4114 N·mm^−2^. The estimated values significantly surpassed the European standard requirements [91] for HDF panels for use in humid conditions (≥2900 N·mm^−2^). Increasing the ALS addition from 6% to 10% resulted in a 29% improvement in MOE values. The incorporation of the increased ALS addition into the UF resin effectively reinforced the composites. The larger ALS quantity in the adhesive acted as a barrier against the influx of water, which resulted in better MOE values. Comparable results, i.e., MOE values ranging from 3730 to 4476 N·mm^−2^, were reported by [6] in their work on the development of eco-friendly, medium-density fiberboards bonded with low phenol-formaldehyde (PF) resin content (3–5%) and calcium lignosulfonate, varying from 5% to 15%, depending on the dry fibers.

Only the HDF panel produced with 3% UF resin and 6% ALS addition content had lower MOE values compared with the control panel (HDF type 4). The HDF panel produced with 10% ALS content had 16% higher MOE values than the control panel.

**Figure 5:**
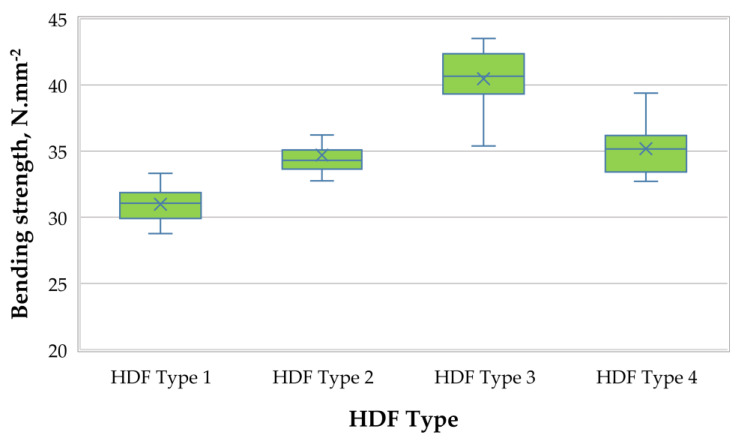
Bending strength (MOR) of HDF panels produced.

A graphical representation of the MOR of the laboratory-produced HDF panels is shown in Figure 5.

The fabricated HDF panels had very satisfactory MOR values, ranging from 30.99 N·mm^−2^ to 40.47 N·mm^−2^, meeting the EN 622-2 standard requirements for HDF panels in humid conditions (MOR ≥ 30 N·mm^−2^) [91]. MOR and MOR of HDF may be affected by a number of factors that are discussed in [93]. According to that study, the fiber dimensions have a significant effect on the physical, mechanical, and thermal properties of HDF panels. The wood species and digester conditions, i.e., press temperature, time, pressure, and defibrator grinding disc distance, are the most important parameters for the fiber quality. Increasing the ALS content from 6% to 10% resulted in improved MOR values by 31%. In the case of MOE, the greater ALS quantity in the adhesive mixture acted as a barrier against the water influx, which resulted in improved MOR values. The panels produced with 6% and 8% ALS content had lower bending strength values than the control panel. The HDF panel fabricated with 10% ALS content had a 27% greater MOR value than the control panel, bonded with 6% UF resin content. The maximum MOR value obtained in this work, i.e., 40.5 N·mm^−2^, was determined at 3% UF resin content and 10% ALS addition. Similar values were reported by Antov et al. (2020) [6] in their work, in which the maximum MOR of 35.2 N·mm^−2^ was recorded for medium-density fiberboards bonded with 5% PF resin and 5% calcium lignosulfonate.

**Figure 6:**
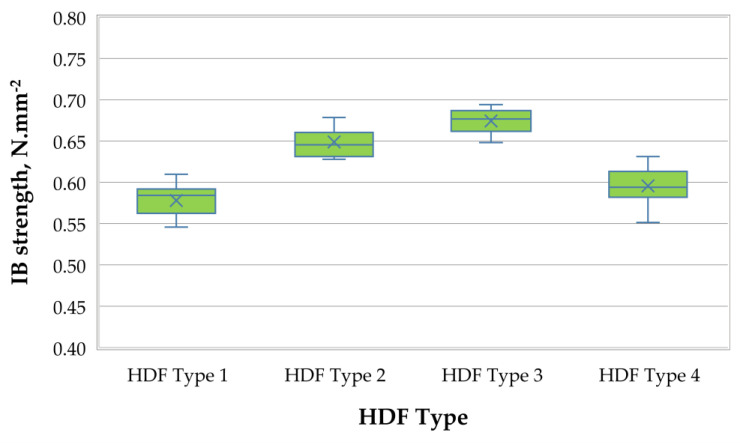
IB strength of HDF panels produced.

Finally, a graphical representation of the average IB strength of the laboratory-produced HDF panels is shown in Figure 6.

The IB strength of the HDF panels ranged from 0.58 N·mm^−2^ to 0.67 N·mm^−2^; this means that increasing the ALS content from 6% to 10% resulted in a 17% increase in IB strength values. A significant improvement, i.e., by 12%, was recorded when the ALS content was increased from 6% to 8%. An increase in ALS significantly improved the interfacial compatibility of the materials. ALS acts as an anionic surfactant, and its molecular structure contains not only polar groups (e.g., hydroxyl and sulfonic groups), but also nonpolar groups (e.g., benzene propane skeleton and aliphatic side chains). With the increase of ALS, its activation gradually reduced the interfacial tension and interfacial free energy, thereby improving the mechanical properties of the composites [94]. There is an apparent correlation between internal bond and thickness swelling; the better a HDF is bonded, the better it resists the forces trying to cause thickness swelling. All laboratory-produced HDF panels met the standard requirements for HDF use in dry conditions (IB ≥ 0.50 N·mm^−2^) [91]. Only the HDF panel bonded with 3% UF resin content and 6% ALS had a lower IB value than the control panel. The laboratory-produced HDF panel, produced with 10% ALS content, had a 13% greater IB strength value compared with the control panel.

### 3.2. Formaldehyde Content

The results for the free formaldehyde content of the fabricated HDF panels, tested in accordance with the standard EN ISO 12460-5 (called the Perforator method), are presented in Table 2.

The results obtained for the free formaldehyde content of the laboratory-produced HDF panels from industrial hardwood fibers, bonded with UF resin and ammonium lignosulfonate adhesive (D-947L), were remarkably low and can be considered as a zero-formaldehyde content [1,24]. All laboratory-fabricated HDF panels fulfilled the requirements of the super E0 emission grade (≤1.5 mg/100 g). The lowest formaldehyde content of 0.7 ± 0.1 mg/100 g was achieved for the HDF panel bonded with 3% UF resin and 10% ALS (D-947L) content. In accordance with the free formaldehyde content results, the reference HDF panel, bonded with 6% UF resin only, can be classified under the emission class E1 (≤8 mg/100 g). These results are in agreement with previous research, where using lignosulfonates as binders for wood composites resulted in decreased free formaldehyde content [6,9,45,69]. ALS has very good characteristics for methylolation due to its large number of phenolic hydroxyl groups and amount of aromatic protons of the guaiacyl units, whose presence tends to increase the reactivity of lignosulfonate toward formaldehyde [94,95].

Natural wood releases low, yet still measurable, amounts of formaldehyde formed by its main polymeric components and extractives at approximately 0.5 to 2 mg/100 g [96,97,98]. Considering this, HDF panels bonded with UF resin and ALS (D-947L) can be defined as extremely low-emission wood-based panels.

## 4. Conclusions

Eco-friendly HDF panels with acceptable physical-mechanical properties and close-to-zero formaldehyde emissions, fulfilling the European standards, can be produced from hardwood fibers bonded with a very low conventional UF resin (3%) and a novel ammonium lignosulfonate at a content of 6% to 10%, depending on the dry fibers. The laboratory-fabricated HDF panels met the stringent standard requirements for use in load-bearing applications in humid conditions. The formaldehyde content of panels produced in the laboratory was distinctly low, ranging from 0.7 mg/100 g to 1.0 mg/100 g (according to EN 12460-5), which is equivalent to the formaldehyde release of natural wood. The HDF panels manufactured with 3% UF gluing content and ammonium lignosulfonate addition >8% exhibited superior physical and mechanical properties compared with those of the control panels produced with a straight UF resin (at 6%). Future studies should focus on decreasing the hot-pressing factor by modifying the formula of ammonium lignosulfonate by adding suitable cross-linking agents, and studying in-depth the bonding interaction among formaldehyde-based resin, lignosulfonate additives, and lignocellulosic fibers.

## Figures and Tables

**Table 1:** Manufacturing parameters used in this research work.

Panel No.	Adhesive Type	Target Density (kg·m^−3^)	UF Resin Content (%)	Ammonium Lignosulfonate Content (%)
1	UF + ALS	910	3	6
2	UF + ALS	910	3	8
3	UF + ALS	910	3	10
4	UF	910	6	0

**Table 2:** Formaldehyde content of HDF panels according to EN ISO 12460-5.

HDF Type	Adhesive	UF Resin Content (%)	Ammonium Lignosulfonate Content (%)	Formaldehyde Content (mg/100 g)
1	UF + ALS	3	6	1.0 ± 0.1
2	UF + ALS	3	8	0.8 ± 0.1
3	UF + ALS	3	10	0.7 ± 0.1
4	UF	6	-	4.3 ± 0.1

## Data Availability

Data sharing is not applicable to this article.

## References

[1] Pizzi A. (2006). Recent developments in eco-efficient bio-based adhesives for wood bonding: Opportunities and issues. J. Adhes. Sci. Technol..

[2] Pizzi A., Papadopoulos A., Policardi F. (2020). Wood Composites and Their Polymer Binders. Polymers.

[3] Taghiyari H.R., Hosseini S.B., Ghahri S., Ghofrani M., Papadopoulos A.N. (2020). Formaldehyde Emission in Micron-Sized Wollastonite-Treated Plywood Bonded with Soy Flour and Urea-Formaldehyde Resin. Appl. Sci..

[4] Ferdosian F., Pan Z., Gao G., Zhao B. (2017). Bio-based adhesives and evaluation for wood composites application. Polymers.

[5] Taghiyari H.R., Esmailpour A., Majidi R., Morrell J.J., Mallaki M., Militz H., Papadopoulos A.N. (2020). Potential Use of Wollastonite as a Filler in UF Resin Based Medium-Density Fiberboard (MDF). Polymers.

[6] Antov P., Savov V., Mantanis G.I., Neykov N. (2020). Medium-density fibreboards bonded with phenol-formaldehyde resin and calcium lignosulfonate as an eco-friendly additive. Wood Mater. Sci. Eng..

[7] Taghiyari H.R., Tajvidi M., Taghiyari R., Mantanis G.I., Esmailpour A., Hosseinpourpia R., Husen A., Jawaid M. (2020). Nanotechnology for Wood Quality Improvement and Protection. Nanomaterials for Agriculture and Forestry Applications.

[8] Ihnát V., Lübke H., Balberčák J., Kuňa V. (2020). Size Reduction Downcycling of Waste Wood—A Review. Wood Res..

[9] Antov P., Mantanis G.I., Savov V. (2020). Development of wood composites from recycled fibres bonded with magnesium lignosulfonate. Forests.

[10] Iždinský J., Vidholdová Z., Reinprecht L. (2020). Particleboards from Recycled Wood. Forests.

[11] Ihnát V., Lübke H., Russ A., Pažitný A., Borůvka V. (2018). Waste agglomerated wood materials as a secondary raw material for chipboards and fibreboards. Part II: Preparation and characterization of wood fibres in terms of their reuse. Wood Res..

[12] Mantanis G., Athanassiadou E., Nakos P., Coutinho A. A New Process for Recycling Waste Fiberboards. Proceedings of the 38th International Wood Composites Symposium.

[13] Barbu M.C., Sepperer T., Tudor E.M., Petutschnigg A. (2020). Walnut and Hazelnut Shells: Untapped Industrial Resources and Their Suitability in Lignocellulosic Composites. Appl. Sci..

[14] Kúdela J. (2020). Surface properties of a medium density fibreboard evaluated from the viewpoint of its surface treatment. Acta Facultatis Xylologiae Zvolen.

[15] Lubis M.A.R., Hong M.K., Park B.D., Lee S.M. (2018). Effects of recycled fiber content on the properties of medium density fiberboard. Eur. J. Wood Prod..

[16] Zhu Z., Buck D., Guo X., Ekevad M., Cao P., Wu Z. (2018). Machinability investigation in turning of high density fiberboard. PLoS ONE.

[17] Kovatchev G. Influence of the Belt Type Over Vibration of the Cutting Mechanism in Woodworking Shaper. Proceedings of the 11th International Science Conference “Chip and Chipless Woodworking Processes”.

[18] Wagner K., Schnabel T., Barbu M.C., Petutschnigg A. (2015). Analysis of selected properties of fiberboard panels manufactured from wood and leather using the near infrared spectroscopy. Int. J. Spectrosc..

[19] Tritchkov N., Antov P. Prospects for Developing the Production of Solid Wood Products Taking into Account the Raw-Material Base. Proceedings of the COST Action E44 Conference “Broad Spectrum Utilisation of Wood”.

[20] Kulman S., Boiko L., Gurová D.H., Sedliačik J. (2019). The Effect of Temperature and Moisture Changes on Modulus of Elasticity and Modulus of Rupture of Particleboard. Acta Facultatis Xylologiae Zvolen.

[21] Savov V., Antov P. (2020). Engineering the Properties of Eco-Friendly Medium Density Fibreboards Bonded with Lignosulfonate Adhesive. Drvna Industrija.

[22] Youngquist J.A. (1999). Wood-Based Composites and Panel Products. Wood Handbook: Wood as an Engineering Material.

[23] Wibowo E.S., Lubis M.A.R., Park B.D., Kim J.S., Causin V. (2020). Converting crystalline thermosetting urea-formaldehyde resins to amorphous polymer using modified nanoclay. J. Ind. Eng. Chem..

[24] Mantanis G.I., Athanassiadou E.T., Barbu M.C., Wijnendaele K. (2018). Adhesive systems used in the European particleboard, MDF and OSB industries. Wood Mater. Sci. Eng..

[25] Kumar R.N., Pizzi A. (2019). Environmental Aspects of Adhesives–Emission of Formaldehyde. Adhesives for Wood and Lignocellulosic Materials.

[26] Dae P.B., Woo K.J. (2008). Dynamic mechanical analysis of urea-formaldehyde resin adhesives with different formaldehyde-to-urea molar ratios. J. Appl. Polym. Sci..

[27] Bekhta P., Sedliacik J., Saldan R., Novak I. (2016). Effect of different hardeners for urea-formaldehyde resin on properties of birch plywood. Acta Fac. Xylologiae Zvolen.

[28] Dunky M., Pizzi A., Mittal K.L. (2003). Adhesives in the Wood Industry. Handbook of Adhesive Technology.

[29] Zhang W., Ma Y., Wang C., Li S., Zhang M., Chu F. (2013). Preparation and properties of lignin-phenol-formaldehyde resins based on different biorefinery residues of agricultural biomass. Ind. Crops Prod..

[30] Jivkov V., Simeonova R., Marinova A., Gradeva G. Study on the Gluing Abilities of Solid Surface Composites with Different Wood Based Materials and Foamed PVC. Proceedings of the 24th International Scientific Conference Wood Is Good–User Oriented Material, Technology and Design.

[31] Kumar R.N., Pizzi A. (2019). Urea-Formaldehyde Resins. Adhesives for Wood and Lignocellulosic Materials.

[32] Tudor E.M., Barbu M.C., Petutschnigg A., Réh R., Krišťák Ľ. (2020). Analysis of Larch-Bark Capacity for Formaldehyde Removal in Wood Adhesives. Int. J. Environ. Res. Public Health.

[33] Tudor E.M., Dettendorfer A., Kain G., Barbu M.C., Réh R., Krišťák Ľ. (2020). Sound-Absorption Coefficient of Bark-Based Insulation Panels. Polymers.

[34] Mirski R., Bekhta P., Dziurka D. (2019). Relationships between Thermoplastic Type and Properties of Polymer-Triticale Boards. Polymers.

[35] Łebkowska M., Radziwiłł M.Z., Tabernacka A. (2017). Adhesives based on formaldehyde—Environmental problems. BioTechnologia.

[36] U.S. Consumer Product Safety Commission (2013). An Update on Formaldehyde (Publication 725).

[37] Bekhta P., Sedliačik J., Noshchenko G., Kačík F., Bekhta N. (2021). Characteristics of Beech Bark and its Effect on Properties of UF Adhesive and on Bonding Strength and Formaldehyde Emission of Plywood Panels. Eur. J. Wood Prod..

[38] World Health Organization (2006). Formaldehyde, 2–Butoxyethanol and 1–tert–Butoxypropan–2–ol. Monographs on the Evaluation of Carcinogenic Risk to Humans.

[39] International Agency for Research on Cancer (2004). IARC Classifies Formaldehyde as Carcinogenic to Humans.

[40] Kawalerczyk J., Siuda J., Mirski R., Dziurka D. (2020). Hemp Flour as a Formaldehyde Scavenger for Melamine-Urea-Formaldehyde Adhesive in Plywood Production. Bioresources.

[41] Papadopoulou E. (2009). Adhesives from renewable resources for binding wood-based panels. J. Environ. Prot. Ecol..

[42] Nordström E., Demircan D., Fogelström L., Khabbaz F., Malmström E. (2017). Green Binders for Wood Adhesives. Applied Adhesive Bonding in Science and Technology.

[43] Hemmilä V., Adamopoulos S., Karlsson O., Kumar A. (2017). Development of sustainable bio-adhesives for engineered wood panels-A review. RSC Adv..

[44] Hosseinpourpia R., Adamopoulos S., Mai C., Taghiyari H.R. (2019). Properties of medium-density fiberboards bonded with dextrin-based wood adhesives. Wood Res..

[45] Antov P., Savov V., Neykov N. (2020). Sustainable Bio-based Adhesives for Eco-Friendly Wood Composites—A Review. Wood Res..

[46] Dunky M. (2020). Wood Adhesives Based on Natural Resources: A Critical Review Part II. Carbohydrate-Based Adhesives, In Reviews of Adhesion and Adhesives.

[47] Sarika P.R., Nancarrow P., Khansaheb A., Ibrahim T. (2020). Bio-Based Alternatives to Phenol and Formaldehyde for the Production of Resins. Polymers.

[48] Park B.D., Kang E.C., Park J.Y. (2008). Thermal curing behavior of modified urea-formaldehyde resin adhesives with two formaldehyde scavengers and their influence on adhesion performance. J. Appl. Polym. Sci..

[49] Costa N., Pereira J., Ferra J., Cruz P., Martins J., Magalhāes F., Mendes A., Carvalho L.H. (2013). Scavengers for achieving zero formaldehyde emission of wood-based panels. Wood Sci. Technol..

[50] Costa N., Pereira J., Martins J., Ferra J., Cruz P., Magalhāes F., Mendes A., Carvalho L. (2012). Alternative to latent catalysts for curing UF resins used in the production of low formaldehyde emission wood-based panels. Int. J. Adhes. Adhes..

[51] de Cademartori P.H.G., Artner M.A., de Freitas R.A., Magalhaes W.L.E. (2019). Alumina nanoparticles as formaldehyde scavenger for urea-formaldehyde resin: Rheological and in-situ cure performance. Compos. B Eng..

[52] Medved S., Gajsek U., Tudor E.M., Barbu M.C., Antonovic A. (2019). Efficiency of bark for reduction of formaldehyde emission from particleboards. Wood Res..

[53] Réh R., Igaz R., Krišt’ák Ľ., Ružiak I., Gajtanska M., Božíková M., Kučerka M. (2019). Functionality of beech bark in adhesive mixtures used in plywood and its effect on the stability associated with material systems. Materials.

[54] Mirski R., Kawalerczyk J., Dziurka D., Siuda J., Wieruszewski M. (2020). The Application of Oak Bark Powder as a Filler for Melamine-Urea-Formaldehyde Adhesive in Plywood Manufacturing. Forests.

[55] Boran S., Usta M., Ondaral S., Gümüskaya E. (2012). The efficiency of tannin as a formaldehyde scavenger chemical in medium density fiberboard. Compos. B Eng..

[56] Bekhta P., Sedliacik J., Kacik F., Noshchenko G., Kleinova A. (2019). Lignocellulosic waste fibers and their application as a component of urea-formaldehyde adhesive composition in the manufacture of plywood. Eur. J. Wood Prod..

[57] Antov P., Savov V., Neykov N. Reduction of Formaldehyde Emission from Engineered Wood Panels by Formaldehyde Scavengers—A Review. Proceedings of the 13th International Scientific Conference Wood EMA 2020 and 31st International Scientific Conference ICWST 2020 Sustainability of Forest-Based Industries in the Global Economy.

[58] Wang Z., Zhao S., Pang H., Zhang W., Zhang S., Li J. (2019). Developing eco-friendly high-strength soy adhesives with improved ductility through multiphase core–shell hyperbranchedpolysiloxane. ACS Sustain. Chem. Eng..

[59] Frihart C., Birkeland M. (2014). Soy Properties and Soy Wood Adhesives.

[60] Zhang B., Zhang F., Wu L., Gao Z., Zhang L. (2020). Assessment of soybean protein-based adhesive formulations, prepared by different liquefaction technologies for particleboard applications. Wood Sci. Technol..

[61] Tan H., Zhang Y., Weng X. (2011). Preparation of the Plywood Using Starch-based Adhesives Modified with blocked isocyanates. Procedia Eng..

[62] Li Z., Wang J., Li C., Gu Z., Cheng L., Hong Y. (2015). Effects of montmorillonite addition on the performance of starch-based wood adhesive. Carbohydr. Polym..

[63] Ndiwe B., Pizzi A., Tibi B., Danwe R., Konai N., Amirou S. (2019). African tree bark exudate extracts as biohardeners of fully biosourced thermoset tannin adhesives for wood panels. Ind. Crops Prod..

[64] Santos J., Antorrena G., Freire M.S., Pizzi A., Álvarez J.G. (2017). Environmentally friendly Wood adhesives based on chestnut (*Castanea sativa*) shell tannins. Eur. J. Wood Wood Prod..

[65] Konai N., Pizzi A., Danwe R., Lucien M., Lionel K.T. (2020). Thermomechanical analysis of African tannins resins and biocomposite characterization. J. Adhes. Sci. Technol..

[66] El Mansouri N.E., Pizzi A., Salvadó J. (2006). Lignin-based wood panel adhesives without formaldehyde. Holz Roh Werkst..

[67] Li R.J., Gutierrez J., Chung Y., Frank C.W., Billington S.L., Sattely E.S. (2018). A lignin-epoxy resin derived from biomass as an alternative to formaldehyde-based wood adhesives. Green Chem..

[68] Gadhave R.V., Srivastava S., Mahanwar P.A., Gadekar P.T. (2019). Lignin: Renewable Raw Material for Adhesive. Open J. Polym. Chem..

[69] Antov P., Jivkov V., Savov V., Simeonova R., Yavorov N. (2020). Structural Application of Eco-Friendly Composites from Recycled Wood Fibres Bonded with Magnesium Lignosulfonate. Appl. Sci..

[70] Lora J.H., Glasser W.G. (2002). Recent industrial applications of lignin: A sustainable alternative to nonrenewable materials. J. Polym. Environ..

[71] Sharma S., Kumar A. (2020). Lignin: Biosynthesis and Transformation for Industrial Applications.

[72] Bajwa D.S., Pourhashem G., Ullah A.H., Bajwa S.G. (2019). A concise review of current lignin production, applications, products and their environmental impact. Ind. Crop Prod..

[73] Klapiszewski Ł., Oliwa R., Oleksy M., Jesionowski T. (2018). Calcium lignosulfonate as eco-friendly additive of crosslinking fibrous composites with phenol-formaldehyde resin matrix. Polymers.

[74] Jin Y., Cheng X., Zheng Z. (2010). Preparation and characterization of phenol–formaldehyde adhesives modified with enzymatic hydrolysis lignin. Bioresour. Technol..

[75] Hemmilä V., Adamopoulos S., Hosseinpourpia R., Sheikh A.A. (2019). Ammonium lignosulfonate adhesives for particleboards with pMDI and furfuryl alcohol as cross-linkers. Polymers.

[76] Vázquez G., González J., Freire S., Antorrena G. (1997). Effect of chemical modification of lignin on the gluebond performance of lignin-phenolic resins. Bioresour. Technol..

[77] Lora J., Belgacem M.N., Gandini A. (2008). Industrial Commercial Lignins: Sources, Properties and Applications. Monomers, Polymers and Composites from Renewable Resources.

[78] Agrawal A., Kaushik N., Biswas S. (2014). Derivatives and applications of lignin—An insight. SciTech J..

[79] Berlin A., Balakshin M., Gupta V.K., Tuohy M., Kubicek C., Saddler J., Xu F. (2014). Industrial Lignins: Analysis, Properties, and Applications. Bioenergy Research: Advances and Applications.

[80] Vishtal A.G., Kraslawski A. (2011). Challenges in industrial applications of technical lignins. BioResources.

[81] Alonso M.V., Oliet M., Rodríguez F., Astarloa G., Echeverría J.M. (2004). Use of a methylolated softwood ammonium lignosulfonate as partial substitute of phenol in resol resins manufacture. J. Appl. Polym. Sci..

[82] Alonso M.V., Oliet M., Rodríguez F., García J., Gilarranz M.A., Rodríguez J.J. (2005). Modification of ammonium lignosulfonate by phenolation for use in phenolic resins. Bioresour. Technol..

[83] EN 310 (1999). Wood-Based Panels-Determination of Modulus of Elasticity in Bending and of Bending Strength.

[84] EN 317 (1998). Particleboards and Fibreboards-Determination of Swelling in Thickness after Immersion in Water.

[85] EN 322 (1998). Wood-Based Panels-Determination of Moisture Content.

[86] EN 323 (2001). Wood-Based Panels-Determination of Density.

[87] EN ISO 12460-5 (2015). Wood-Based Panels-Determination of Formaldehyde Release—Part 5. Extraction Method (Called the Perforator Method).

[88] Kordkheili H.Y., Najafi S.K., Eshkiki R.B., Pizzi A. (2015). Improving urea formaldehyde resin properties by glyoxalated soda bagasse lignin. Eur. J. Wood. Prod..

[89] Kim J.W., Carlbom K., Matuana L., Heiden P. (2006). Thermoplastic modification of urea-formaldehyde wood adhesives to improve moisture resistance. J. Appl. Polym. Sci..

[90] Mihajlova J., Savov V. Physical Indicators of High-Density Fibreboards (HDF) Manufactured from Wood of Hard Broadleaved Species. Proceedings of the 8th Hardwood Conference.

[91] EN 622-2 (2004). Fibreboards–Specifications—Part 2: Requirements for Hardboard.

[92] Shi S.Q., Gardner D.J. (2006). Hygroscopic thickness swelling rate of compression molded wood fiberboard and wood fiber/polymer composites. Compos. Part A Appl. Sci. Manuf..

[93] Nasir M., Khali D.P., Jawaid M., Tahir P.M., Siakeng R., Asim M., Khan T.A. (2019). Recent development in binderless fiber-board fabrication from agricultural residues: A review. Constr. Build. Mater..

[94] Hu J.P., Guo M.H. (2015). Influence of ammonium lignosulfonate on the mechanical and dimensional properties of wood fiber biocomposites reinforced with polyactic acid. Ind. Crops Prod..

[95] Alonso M.V., Rodriguez J.J., Oliet M., Rodriguez F., Garcia J., Gilarranz M.A. (2001). Characterization and Structural Modification of Ammonic Lignosulfonate by Methylolation. J. Appl. Polym. Sci..

[96] Roffael E. (2006). Volatile organic compounds and formaldehyde in nature, wood and wood based panels. Holz Roh Werkst..

[97] Athanassiadou E., Roffael E., Mantanis G. Medium Density Fiberboards (MDF) from Recycled Fibres. Proceedings of the Conference “Towards a Higher Technical, Economical and Environmental Standard in Europe” COST Action E31.

[98] Salem M.Z.M., Böhm M. (2013). Understanding of formaldehyde emissions from solid wood: An overview. BioResources.

